# Four-Octyl Itaconate Attenuates UVB-Induced Melanocytes and Keratinocytes Apoptosis by Nrf2 Activation-Dependent ROS Inhibition

**DOI:** 10.1155/2022/9897442

**Published:** 2022-03-11

**Authors:** Yongyi Xie, Zile Chen, Zhouwei Wu

**Affiliations:** Department of Dermatology, Shanghai General Hospital, Shanghai Jiao Tong University School of Medicine, Shanghai 200080, China

## Abstract

Vitiligo is an acquired skin depigmentation disease in which excessive reactive oxygen species (ROS) play a critical pathogenic role in melanocyte destruction. The complex crosstalk between melanocytes and keratinocytes in vitiligo suggests that treatments aimed at protecting both the cells might be meaningful. In this study, we investigated the effect of 4-octyl itaconate (4-OI), an itaconate derivative, on ultraviolet B- (UVB-) induced apoptosis in HaCaT and PIG1 cells and the underlying mechanisms. HaCaT and PIG1 cells were pretreated with 4-OI (50 or 100 *μ*M) for 24 h and then exposed to 300 mJ/cm^2^ UVB (emission range 290–320 nm, emission peak 310 nm). ROS levels and cell apoptosis were investigated using fluorescence microscopy and flow cytometry 24 h after irradiation. In addition, nuclear translocation and the expression of pathway-related proteins and mRNAs were detected using confocal microscopy, western blotting, and qRT-PCR, respectively. Our results demonstrated that UVB induced apoptosis in HaCaT and PIG1 cells, whereas inhibition of ROS production could reverse this effect. Furthermore, 4-OI attenuated UVB-induced apoptosis in HaCaT and PIG1 cells in a concentration-dependent manner by reducing the ROS levels. Moreover, 4-OI induced nuclear translocation and activation of nuclear factor erythroid 2-related factor 2 (Nrf2), and Nrf2 silencing reversed the inhibitory effect of 4-OI on the UVB-induced increase in ROS production and apoptosis in HaCaT and PIG1 cells. In addition, *in vivo* experiments using the Institute of Cancer Research mouse model showed that 4-OI *via* tail vein injection (10 mg/kg/day for six consecutive days) could reduce skin damage induced by UVB (400 mJ/cm^2^/day for five consecutive days). In conclusion, 4-OI can protect melanocytes and keratinocytes from UVB-induced apoptosis by Nrf2 activation-dependent ROS inhibition and can potentially treat skin disorders associated with oxidative stress, such as vitiligo.

## 1. Introduction

Vitiligo is a chronic, acquired disorder characterized by skin depigmentation, with an estimated global prevalence of 0.5%–1.0% [1]. Several factors such as environmental stimuli, gene susceptibility, autoimmune dysregulation, and oxidative stress are associated with vitiligo development [2, 3]. In addition, many studies, including our previous study, have recently revealed the critical role of oxidative stress in vitiligo pathogenesis [4–7].

It is widely accepted that ultraviolet B (UVB) irradiation (290–320 nm) can lead to excessive reactive oxygen species (ROS) production and cell apoptosis and may trigger vitiligo flare-up [2, 8, 9]. Aberrant ROS generation caused by UVB irradiation can lead to melanocyte stress, promote protein oxidation and carbonylation, and result in melanocyte dysfunction [10]. Interestingly, growing evidence suggests a complex link between keratinocytes and melanocytes under oxidative stress conditions. A study reported that keratinocytes released extracellular adenosine 5′-triphosphate (ATP) after oxidative stress and subsequently induced ROS generation and death in melanocytes [11]. Moreover, a previous study revealed the impaired mitochondrial function of keratinocytes in vitiligo [12, 13]. These structural defects might hinder the ability of keratinocytes to maintain melanocyte homeostasis, resulting in melanocyte damage.

Nuclear factor erythroid 2-related factor 2 (Nrf2) is a ubiquitously expressed transcription factor that regulates cellular responses to oxidative stress [14]. Normally, Nrf2 is combined with Kelch-like ECH-associated protein 1 (Keap1) to form a complex. Oxidative injury and other stress conditions could lead to keap1-Nrf2 dissociation [15]. Then, Nrf2 translocate from the cytoplasm into the nucleus and activate antioxidative genes such as heme oxygenase-1 (HO-1) and NAD(P)H:quinone oxidoreductase 1 (NQO1), which could generate cytoprotective effects [16]. HO-1 maintains cellular redox homeostasis as a major effector protein [17]. Interestingly, previous studies showed that Nrf2 signaling was impaired in vitiligo, and thus, ROS could not be effectively scavenged [5, 18]. Thus, medications that could activate Nrf2 signaling and reduce ROS accumulation in both keratinocytes and melanocytes may benefit from vitiligo therapy.

Itaconate is a metabolite produced by the tricarboxylic acid (TCA) cycle and has multiple immunoregulatory properties, including anti-inflammatory and antioxidative effects [19, 20]. A cell-permeable itaconate derivative, 4-octyl itaconate (4-OI), has been shown to exert protective effects in many disease models. For example, Yi et al. [21] demonstrated that 4-OI treatment protected hepatocytes from liver ischemia-reperfusion damage by reducing oxidative stress. Similarly, 4-OI can also reduce H_2_O_2_-induced ROS production and death in neuronal cells [22]. However, the effects of 4-OI on UVB-induced skin cell damage have not been elucidated.

Given that both melanocytes and keratinocytes play important roles in vitiligo [2, 23, 24], the present study is aimed at investigating the protective effects and possible underlying mechanisms of 4-OI in UVB-induced responses *in vitro*, using HaCaT and PIG1 cells, and *in vivo*, using a mouse model of skin damage. Our findings highlight 4-OI as a potential therapeutic option for vitiligo.

## 2. Material and Methods

### 2.1. Cell Culture and Treatment

HaCaT cells (ATCC, MD, USA) and PIG1 cells (ATCC, MD, USA) were cultured in Dulbecco's modified Eagle's medium (DMEM; Gibco, Grand Island, NY, USA) supplemented with 10% fetal bovine serum (FBS) and 1% antibiotic-antimycotic (Gibco, Grand Island, NY, USA) at 37°C and 5% CO_2_. The cells were seeded in 12-well culture plates, allowed to attach for 24 h, and subsequently treated with 4-OI or exposed to UVB. Control cultures received a medium containing a comparable DMSO concentration (up to 0.05%). In addition, fresh medium containing 4-OI (MedChemExpress, USA), N-acetyl-l-cysteine (NAC, MedChemExpress, USA), or DMSO was added as indicated in the figure legends.

### 2.2. UVB Exposure

UVB treatment of cells was performed using Waldmann UV109B lights with TL-12 lamps (Waldmann Lighting Ltd., Germany) emitting primarily in the 290–320 nm range, with an emission peak at 310 nm, as described by Wu et al. [25]. Cells were irradiated in phosphate-buffered saline (PBS) with Mg^2+^/Ca^2+^ at a distance of 40 cm. The UVB dose used in this study is indicated in the figure legends. Sham-irradiated cells were handled similarly but shielded with an aluminum foil against UVB exposure. The PBS was removed after irradiation, culture medium was added, and cells were cultured as indicated in the figure legends.

### 2.3. Assessment of Cell Viability

Cell Counting Kit-8 (CCK-8) (Yeasen, Shanghai, China) was used to assess cell viability. HaCaT and PIG1 cells were cultured in 96-well plates. Twenty-four hours after treatment, 10 *μ*L CCK-8 reagent was added to each well, followed by incubation for another 4 h. The absorbance of each sample was measured at 450 nm wavelength using a spectrophotometer (Thermo Fisher Scientific Oy, Vantaa, Finland).

### 2.4. Apoptosis Determination

Apoptosis assays were performed using the Annexin V-fluorescein isothiocyanate (FITC)/propidium iodide (PI) double staining kit (Dojindo, Shanghai, China), according to the manufacturer's instructions. HaCaT and PIG1 cells were treated as indicated in the figure legend. The cells were then collected, resuspended in 100 *μ*L binding buffer, and incubated with Annexin V-FITC/PI in the dark for 15 min. Subsequently, the samples were diluted with 400 *μ*L of binding buffer before examination by flow cytometry. The apoptotic cell populations included early apoptotic (Q3) and late apoptotic (Q2) cells [26].

### 2.5. Detection of Intracellular ROS Production

The commonly used fluorescent probe 2′, 7′-dichlorofluorescein diacetate (DCFH-DA) was used to determine intracellular ROS levels. HaCaT and PIG1 cells were seeded in 6-well plates at densities of 1 × 10^5^ and 5 × 10^4^ cells/mL, respectively. After treatment, 10 *μ*M DCFH-DA (Beyotime) was added to each well, and the plate was incubated at 37°C for 30 min. The cells were observed and photographed using an inverted fluorescence microscope (Leica, Wetzlar, Germany). In addition, the DCFH-DA fluorescence intensity was measured using Accuri C6 flow cytometry (Accuri, Ann Arbor, MI, USA) at 488 nm excitation and 525 nm emission. The results were analyzed using FlowJo software (version 10.0).

### 2.6. Quantitative Reverse Transcription-Polymerase Chain Reaction (qRT-PCR)

*NRF2*, *HO-1*, and *ACTB* mRNA levels were measured using qRT-PCR. First, total RNA was extracted using the RNAiso Plus Reagent (Invitrogen, Carlsbad, CA, USA). Thereafter, reverse transcription was performed using the PrimeScript RT-PCR Kit (Takara Bio, Japan). qRT-PCR was performed on an Mx3000p real-time system (Stratagene, USA) using SYBR Premix Ex Taq (Yeasen, Shanghai, China). Amplification was initiated at 95°C for 30 s as the first step, followed by 40 successive cycles of 95°C for 5 s and 60°C for 20 s. Primers from Takara Bio are listed in Supplementary Table 1.

### 2.7. Western Blotting Analysis

HaCaT and PIG1 cells were lysed in RIPA lysis buffer on ice. Equal amounts of protein from each sample were separated using 10% SDS-PAGE and then transferred to polyvinylidene fluoride membranes (Merck Millipore, Billerica, MA, USA). In addition, 5% bovine serum albumin (BSA) (Yeasen, Shanghai, China) was used to block nonspecific binding for 1 h. The membranes were probed with anti-Keap1 (ab227828), anti-Nrf2 (ab62352), anti-HO-1 (ab13248), and anti-*β*-actin (ab8227) antibodies overnight at 4°C; these primary antibodies were obtained from Abcam (Cambridge, MA, USA). Membranes were then incubated with secondary antibodies, including goat anti-mouse (115-035-003; Jackson ImmunoResearch, PA, USA) and goat anti-rabbit (111-035-003, Jackson ImmunoResearch, PA, USA) antibodies, at room temperature for 1 h. Thereafter, the protein bands were examined using an ECL western blot detection system (Merck Millipore, Burlington, MA, USA). The antibodies used are listed in Supplementary Table 2.

### 2.8. Immunofluorescence

After treatment, the HaCaT and PIG1 cells were washed with PBS-Tween 20 (PBST) and fixed with 4% paraformaldehyde for 20 min. The samples were then permeabilized with 0.3% Triton X-100 for 20 min and blocked with 1% BSA for 30 min. Cells were then incubated with a primary anti-Nrf2 antibody (ab62352, Abcam, Cambridge, MA, USA) overnight at 4°C. The samples were incubated with an iFluor™ 488 antibody (Cat#16608, AAT Bioquest, Sunnyvale, CA, USA) at room temperature for 1 h. Samples were washed three times with PBST, and DAPI (Yeasen, Shanghai, China) was used to stain the nuclei. Finally, the samples were photographed using a TCS SP8 X confocal microscope (Leica, Mannheim, Germany).

### 2.9. Transfection with Nrf2-Targeted Specific Small Interfering RNA (siRNA)

siRNA targeting Nrf2 (si-Nrf2, s9492) and siRNA consisting of a scrambled sequence not leading to the specific degradation of any cellular message (si-control) were purchased from Ambion (Austin, TX, USA). Cells were treated with a mixture of Opti-MEM, Lipofectamine 3000 (Invitrogen, Carlsbad, CA, USA), and si-Nrf2 (10 nM). After a 24 h incubation period, siRNA-transfected cells were used for further experiments. siRNA transfection did not affect cell viability, as demonstrated by microscopy (data not shown).

### 2.10. Animal Experiments

The Institute of Cancer Research (ICR) mice are used to establish the UVB-irradiated animal model [27, 28]. ICR mice (6–8 weeks old, body weight 20-25 g, Charles River Laboratories, Shanghai) were fed a standard laboratory diet and water and maintained under a 12 h light/dark cycle (40%–50% humidity, 25°C). Mice were randomly divided into three groups: control group (*n* = 5), UVB group (*n* = 5), and UVB+4-OI group (*n* = 5). In the UVB group, the backs of mice were exposed to UVB irradiation (400 mJ/cm^2^) once a day for five consecutive days. In the UVB+4-OI group, 4-OI was dissolved in (2-hydroxypropyl)-*β*-cyclodextrin in phosphate-buffered saline (PBS). Mice were subjected to tail vein injection with a final volume of 100 *μ*L 4-OI (10 mg/kg) every day for six consecutive days, and UVB irradiation (400 mJ/cm^2^) was started on the second day of 4-OI administration, for five consecutive days. Mice were sacrificed 24 h after the last UVB treatment. A constant area (1 × 1 cm) from the dorsal skin of the mice was weighed. The experimental protocol was approved by the Institutional Animal Care and Use Committee of Shanghai General Hospital (2021AW006).

### 2.11. Hematoxylin and Eosin (H&E) and TUNEL Staining

The dorsal skin of the mice was fixed in buffered formaldehyde solution. Paraffin-embedded tissues were sliced into five-micrometer-thick sections for H&E staining. TUNEL staining was performed using a FragEL™ DNA Fragmentation Detection Kit (Merck, Germany) with DAB (GeneTech, Shanghai, China) according to the manufacturer's instructions. Images were captured using a DM5500 B Leica microscope.

### 2.12. Statistical Analysis

The data are presented as mean ± SD and were analyzed using one-way ANOVA followed by Brown-Forsythe and Welch ANOVA tests or unpaired Student's *t*-test. Analyses were carried out using GraphPad Prism software (version 8.0; San Diego, CA, USA). Statistical significance was defined as *P* < 0.05.

## 3. Results

### 3.1. UVB-Induced Cytotoxicity and Apoptosis Are Inhibited by 4-OI in a Concentration-Dependent Manner in HaCaT and PIG1 Cells

**Figure 1:**
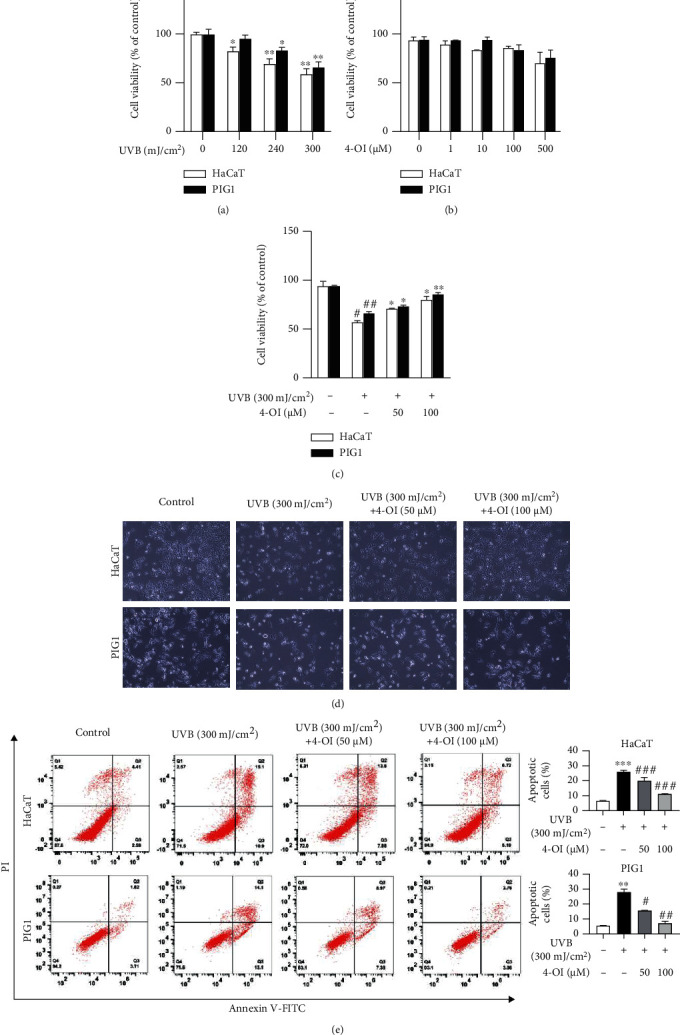
4-OI inhibited UVB-induced cytotoxicity and apoptosis in a concentration-dependent manner in HaCaT and PIG1 cells. (a) HaCaT and PIG1 cells were exposed to different doses of UVB (0, 120, 240, and 300 mJ/cm^2^) and investigated 24 h postirradiation. (b) HaCaT and PIG1 cells were treated with different concentrations of 4-OI (0, 1, 10, 100, and 500 *μ*M) for 24 h. (c) HaCaT and PIG1 cells were pretreated with 4-OI (50 and 100 *μ*M) for 24 h, then exposed to 300 mJ/cm^2^ UVB, and investigated 24 h postirradiation. The cell viability was determined by CCK-8 cell viability assay. Cellular viability rate and statistical significance were determined with respect to the 100% viability of untreated control cells. HaCaT and PIG1 cells were exposed to UVB (300 mJ/cm^2^) with or without pretreatment with 4-OI (50 and 100 *μ*M) and investigated 24 h postirradiation. (d) Morphological changes. Magnification: ×20. (e) Cell apoptosis by flow cytometry. Data were presented as means ± SD (*n* = 3) of one representative experiment of three. ^∗^*P* < 0.05, ^∗∗^*P* < 0.01, and ^∗∗∗^*P* < 0.001 versus the control group; ^#^*P* < 0.05, ^##^*P* < 0.01, and ^###^*P* < 0.001 versus the UVB group.

HaCaT and PIG1 cells were exposed to UVB (0, 120, 240, and 300 mJ/cm^2^) or 4-OI (0, 1, 10, 100, and 500 *μ*M) for 24 h, and the cell viability was examined. UVB inhibited cell viability in a dose-dependent manner, whereas 4-OI did not affect cell viability up to 500 *μ*M in HaCaT and PIG1 cells (Figures 1(a) and 1(b)). Thus, UVB irradiation at 300 mJ/cm^2^ and different concentrations of 4-OI (50 and 100 *μ*M) were chosen for further experiments. To investigate the protective effect of 4-OI on UVB-induced inhibition of cell viability, HaCaT and PIG1 cells were pretreated with 50 or 100 *μ*M 4-OI for 24 h before UVB irradiation (300 mJ/cm^2^). The results showed that 4-OI reversed the UVB-induced inhibition of cell viability in a concentration-dependent manner in both cell lines (Figure 1(c)). We also showed that pretreatment with 4-OI attenuated the morphological changes caused by UVB irradiation in HaCaT and PIG1 cells (Figure 1(d)).

UVB is known to induce cell apoptosis; thus, we examined whether 4-OI exerted a protective effect against UVB-induced cell apoptosis. As shown in Figure 1(e), UVB exposure (300 mJ/cm^2^) significantly increased the proportion of apoptotic cells (Q3 + Q2, 26%) compared to that in the control (Q3 + Q2, 7%), whereas pretreatment with 4-OI (50 and 100 *μ*M) rescued UVB-induced cell apoptosis (Q3 + Q2) to 21.7% and 11.9%, respectively, in HaCaT cells. Similarly, UVB-induced PIG1 cell apoptosis (Q3 + Q2, 27.2%) was inhibited by 4-OI pretreatment in a concentration-dependent manner (Q3 + Q2, 16.3% and 6.7%, respectively). These data suggest that 4-OI protects HaCaT and PIG1 cells from UVB-induced damage.

### 3.2. UVB Induced Cell Apoptosis through ROS Production

**Figure 2:**
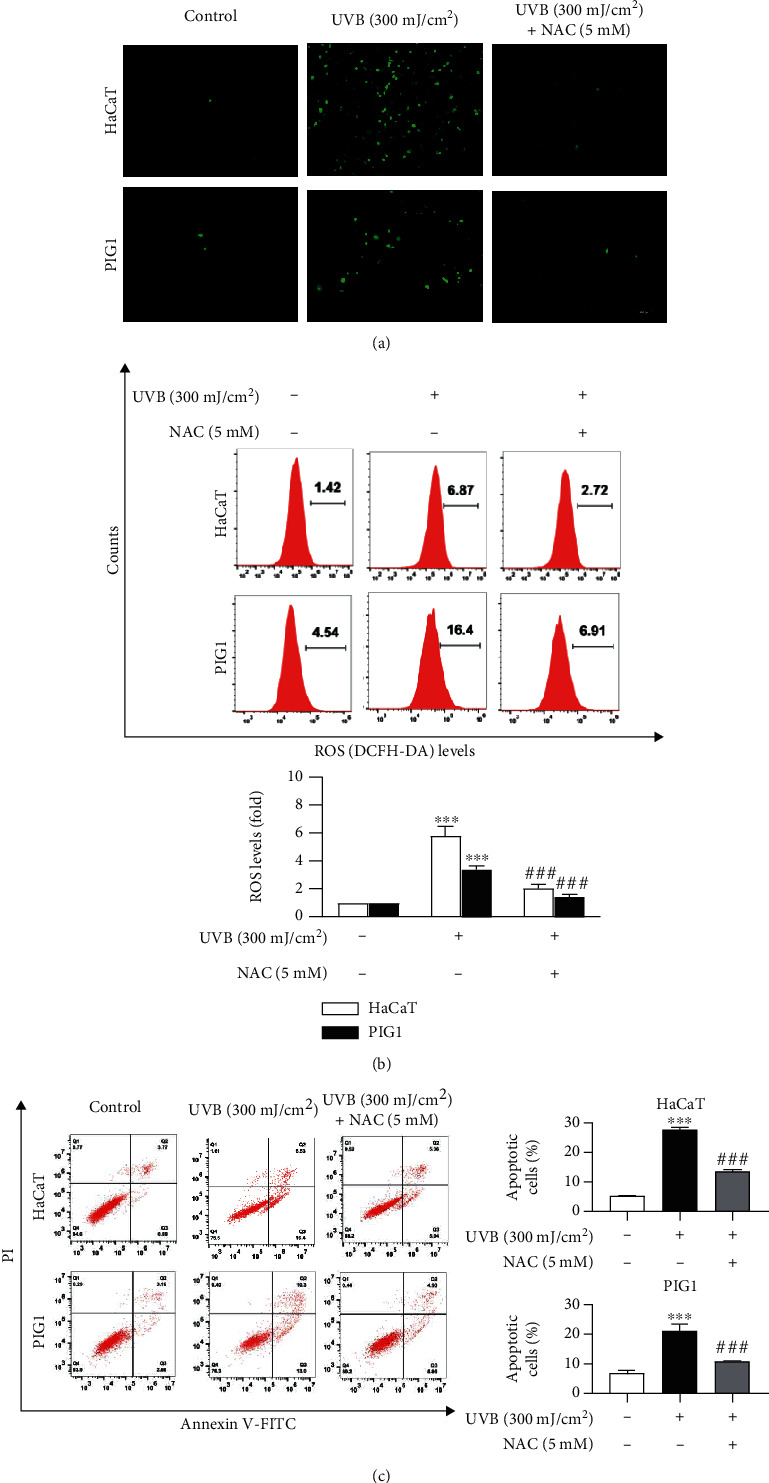
UVB induced cell apoptosis through ROS production. HaCaT and PIG1 cells were exposed to UVB (300 mJ/cm^2^) with or without 1 h pretreatment with NAC (5 mM). Twenty-four hours after stimulation, cells were incubated with DCFH-DA, and the level of ROS production was detected by (a) fluorescence microscopy (scale bar = 100 *μ*m) and (b) flow cytometric analysis. (c) Apoptosis of HaCaT and PIG1 cells was investigated 24 h postirradiation by flow cytometry. Data are expressed as mean ± SD (*n* = 3) of one representative experiment of three. ^∗∗∗^*P* < 0.001 versus the control group; ^###^*P* < 0.001 versus the UVB group.

A previous study showed that UVB irradiation might induce skin cell apoptosis *via* excessive oxidative stress [10]. To further explore the role of ROS production in UVB-induced apoptosis in HaCaT and PIG1 cells, these cells were incubated with or without NAC (5 mM), an ROS scavenger, for 1 h before exposure to 300 mJ/cm^2^ UVB. Intercellular ROS generation was detected using fluorescence imaging and flow cytometry assays. We demonstrated that UVB mediated excess ROS generation in both the cell lines, whereas the addition of NAC (5 mM) significantly blocked UVB-induced ROS production (Figures 2(a) and 2(b)). Consistently, apoptosis analysis showed that NAC (5 mM) pretreatment reduced UVB-induced HaCaT cell apoptosis (11.3%) compared to that in the UVB irradiation group (22.9%). Furthermore, pretreatment of PIG1 cells with NAC (5 mM) for 1 h markedly decreased UVB-mediated apoptosis (Figure 2(c)). These data confirm the role of ROS production in UVB-mediated cell apoptosis.

### 3.3. Nuclear Translocation of Nrf2 and Nrf2/HO-1 Pathway Activation Was Promoted by 4-OI

**Figure 3:**
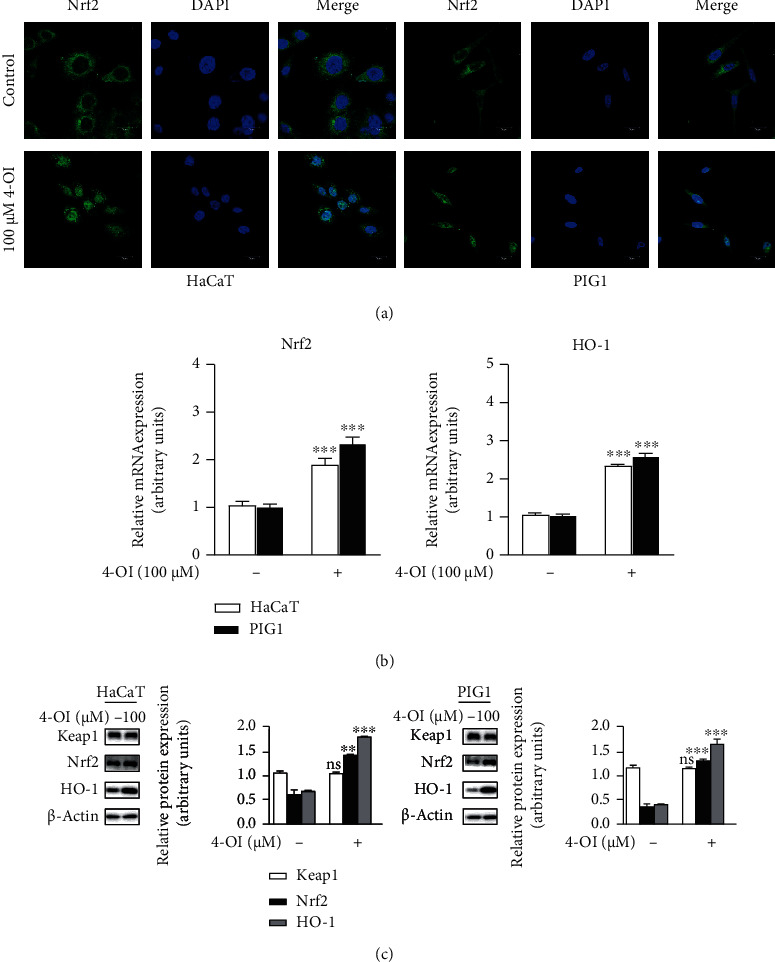
4-OI could promote Nrf2 nuclear translocation and activate the Nrf2/HO-1 pathway. (a) HaCaT and PIG1 cells were treated with 4-OI (100 *μ*M) for 3 h; then, the distribution of Nrf2 was observed by confocal laser scanning microscopy (scale bar = 25 *μ*m). HaCaT and PIG1 cells were treated with 4-OI (100 *μ*M) for 24 h for (b) qPCR analysis and (c) western blotting and quantitative analysis. Nrf2 and HO-1 mRNA levels were normalized for *β*-actin mRNA levels. Keap1, Nrf2, and HO-1 protein levels were normalized to *β*-actin protein levels by ImageJ software. Results are expressed as mean ± SD (*n* = 3) of one representative experiment of at least three. ^∗∗∗^*P* < 0.001 versus the control group.

The Nrf2/HO-1 signaling pathway plays an important role in regulating oxidative stress [29]. Therefore, we first investigated whether 4-OI could induce Nrf2 translocation from the cytoplasm to the nuclei of HaCaT and PIG1 cells. The cells were incubated with 100 *μ*M 4-OI for 3 h. Immunofluorescence staining illustrated that 4-OI promoted the translocation of Nrf2 from the cytoplasm to the nucleus in HaCaT and PIG1 cells (Figure 3(a)). As expected, mRNA expression of *Nrf2* and its downstream factor *HO-1* increased significantly in the two cell lines after 24 h of treatment with 100 *μ*M 4-OI (Figure 3(b)). Furthermore, whole-cell Nrf2 and HO-1 protein expression levels were consistently increased by treatment with 4-OI in HaCaT and PIG1 cells (Figure 3(c)). In addition, Keap1 protein expression was comparable with or without the treatment of 4-OI (Figure 3(c)).

### 3.4. UVB-Induced Cell Apoptosis Was Rescued by 4-OI via Nrf2/HO-1 Activation-Dependent ROS Inhibition

**Figure 4:**
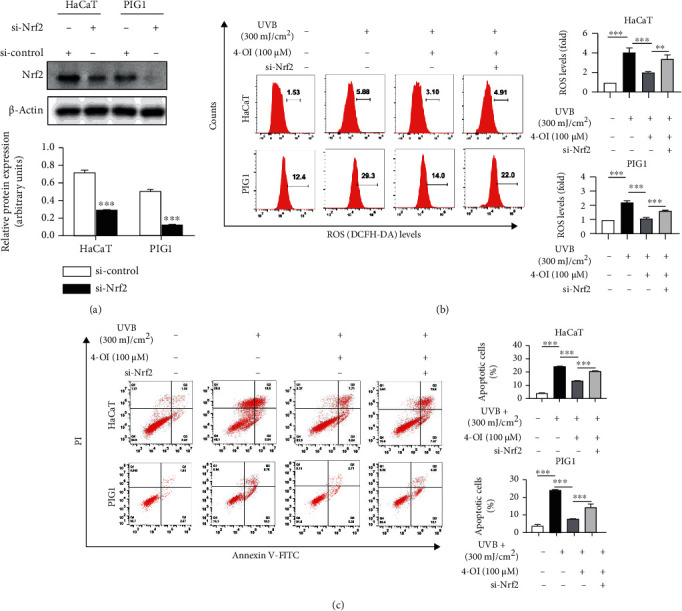
4-OI rescued UVB-induced cell apoptosis via Nrf2/HO-1 activation-dependent ROS inhibition. (a) Western blotting analysis for the effect of si-Nrf2 transfection in HaCaT and PIG1 cells. si-control or si-Nrf2-transfected cells were pretreated with 4-OI (100 *μ*M) for 24 h prior to UVB exposure (300 mJ/cm^2^); then, the ROS levels (b) and apoptosis (c) were determined by flow cytometry. Results are expressed as mean ± SD (*n* = 3) of one representative experiment of at least three. ^∗^*P* < 0.05, ^∗∗^*P* < 0.01, and ^∗∗∗^*P* < 0.001 versus the control group.

To determine whether the protective effect of 4-OI on UVB-induced cell damage was mediated by Nrf2 signaling, we transfected HaCaT and PIG1 cells with si-Nrf2 to silence *Nrf2*. As shown in Figure 4(a), the expression of Nrf2 was successfully knocked down at the protein level in HaCaT and PIG1 cells. Subsequently, HaCaT and PIG1 cells were incubated with 100 *μ*M 4-OI for 24 h before UVB exposure, and intracellular ROS generation was detected using flow cytometry. As expected, 100 *μ*M 4-OI significantly reduced UVB-induced ROS generation in HaCaT and PIG1 cells, whereas these effects were abrogated in the si-Nrf2-transfected cells (Figure 4(b)). Furthermore, 4-OI pretreatment attenuated UVB-induced apoptosis in HaCaT cells. However, in the si-Nrf2 condition, the apoptotic cell population was comparable to that in the UVB-treated HaCaT cells. Our results also revealed that Nrf2 knockdown markedly suppressed the inhibitory effects of 4-OI on UVB-induced apoptosis in PIG1 cells (Figure 4(c)).

### 3.5. Mouse Skin Was Protected by 4-OI from UVB-Induced Damage

**Figure 5:**
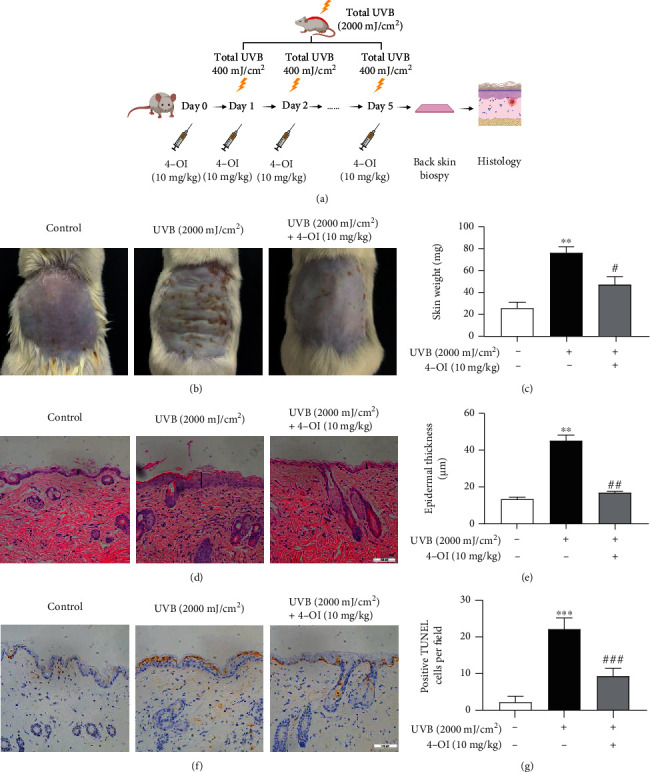
4-OI protected the skin of the mouse model from UVB-induced damage. (a) In the UVB group, the back of mice was exposed to UVB irradiation (400 mJ/cm^2^) once a day for 5 consecutive days. In the UVB with 4-OI group, mice were subjected to tail vein injection with 4-OI (10 mg/kg) every day for 6 consecutive days. UVB irradiation (400 mJ/cm^2^) started from the second day of 4-OI administration and for 5 consecutive days. All mice were sacrificed 24 h following the last UVB treatment. (b) The changes of mouse dorsal skin 24 h following the last UVB treatment with or without 4-OI treatment. (c) A constant skin area (1 × 1 cm) of mouse dorsal back exposed to UVB was weighed. (d) Histopathological examination of skin area exposed to UVB exposure. The black lines represented epidermis thickness. Hematoxylin and eosin staining, scale bar = 100 *μ*m. (e) Epidermis thicknesses in each group were analyzed. Cell apoptosis in the epidermis was analyzed by (f) TUNEL staining and (g) analysis, scale bar = 100 *μ*m. Data were presented as means ± SD (*n* = 5). ^∗∗^*P* < 0.01 versus the control group; ^#^*P* < 0.05 and ^##^*P* < 0.01 versus the UVB group.

To investigate the protective role of 4-OI on the skin *in vivo*, the backs of mice were exposed to UVB irradiation (400 mJ/cm^2^) once a day for five consecutive days with or without pretreatment with tail vein injection of 4-OI (10 mg/kg) once a day for six days (Figure 5(a)). The results showed that UVB treatment led to scaling and thickening of the dorsal back skin in UVB-exposed mice, while pretreatment with 4-OI ameliorated UVB-induced skin changes in mice (Figure 5(b)). Moreover, after UVB irradiation, the skin weight increased approximately threefold compared to the control mice. However, 4-OI pretreatment significantly reduced the UVB-induced increase in skin weight (Figure 5(c)). Furthermore, histopathological examination revealed that the epidermis was thicker in the UVB-irradiated group than that in the control group. However, 4-OI pretreatment caused a significant decrease in the thickness of the epidermis thickened by UVB exposure (Figures 5(d) and 5(e)). In addition, we observed that UVB irradiation markedly increased the number of TUNEL-positive cells in the epidermis, whereas 4-OI pretreatment inhibited this effect (Figures 5(f) and 5(g)). These findings indicate that 4-OI protects against UVB-induced skin damage in the mouse epidermis *in vivo*.

## 4. Discussion

**Figure 6:**
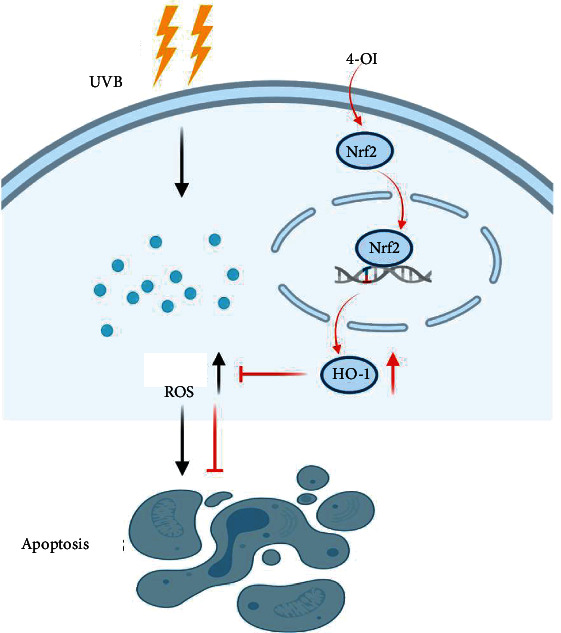
A proposed mechanism of the protective effect of 4-OI in UVB-induced skin cell damage. UVB causes HaCaT and PIG1 cell apoptosis via ROS production. 4-OI promotes Nrf2 nuclear translocation, activates the Nrf2/HO-1 signaling pathway, subsequently inhibits ROS generation, and then alleviates cell apoptosis.

Although melanocyte destruction is a key event in vitiligo, it has been reported that keratinocytes from vitiligo patients also suffer from oxidative stress and apoptotic features [24], indicating that the entire epidermis is involved in the development of vitiligo. Thus, therapies aimed at protecting both melanocytes and keratinocytes might be useful in treating vitiligo. In this study, we reported that 4-OI, a cell-permeable derivative of itaconate, mitigated UVB-induced oxidative stress and apoptosis in both HaCaT and PIG1 cells by activating Nrf2/HO-1 signaling (Figure 6). Moreover, our *in vivo* study showed that 4-OI relieved UVB-induced skin damage in animal models.

UVB exposure is considered one of the most important triggers of vitiligo [2, 25]. Several lines of evidence have illustrated that UVB leads to inflammation and apoptosis by elevating ROS production and subsequent oxidative damage [9, 18, 30]. We previously reported that UVB increases ROS production and causes inflammatory cytokine release in keratinocytes [25]. In this study, we reaffirmed that UVB could cause both keratinocyte and melanocyte apoptosis through increased ROS production.

Several studies have shown that oxidative stress plays an important role in melanocyte damage [12, 31]. An imbalance in the antioxidant system and overloaded ROS generation cause melanocyte destruction through direct or indirect mechanisms [32]. In contrast, ROS can activate the caspase pathway, promote the release of interleukins 1*β* and 18, and then cause innate immune cell activation. The immune response finally results in melanocyte destruction. Conversely, impaired SIRT3 in vitiligo melanocytes results in their apoptosis by inducing mitochondrial dynamic remodeling and oxidative stress [3]. In addition, the role of ROS in keratinocytes in the pathogenesis of vitiligo has been a focus of research. Li et al. [6] showed that ROS-induced keratinocytes release CXCL16, which can mediate CXCR6^+^CD8^+^ T-cell skin trafficking and then initiate an adaptive immune response that causes melanocyte damage. Ahn *et al.* [11] showed that stressed keratinocytes could induce ROS generation and death in melanocytes by releasing ATP into the extracellular regions. Our results showed that 4-OI pretreatment decreased UVB-induced ROS production and subsequent apoptosis in a concentration-dependent manner in HaCaT and PIG1 cells. Meanwhile, the ROS inhibition and antiapoptotic property of 4-OI were comparable to NAC, a standard antioxidant. These findings suggest that 4-OI can protect melanocytes and keratinocytes from UVB in vitiligo.

Recently, itaconate was reported as a novel and promising Nrf2 activator that promotes the dissociation of Keap1 from Nrf2, leading to Nrf2 nuclear translocation [33]. Liu et al. [22] have revealed that 4-OI protects neuronal cells from oxidative damage by activating Keap1-Nrf2 signaling. Moreover, Tang *et al.* [34] have found that 4-OI causes sustained activation and nuclear translocation of Nrf2 to inhibit ROS generation in human umbilical vein endothelial cells. Consistent with these findings, we found that 4-OI promoted nuclear translocation of Nrf2 and increased HO-1 expression in HaCaT and PIG1 cells. Therefore, we inferred that the protective effects of 4-OI on UVB-induced cell damage might be exerted by reducing ROS generation through a possible signaling pathway, Nrf2/HO-1 signaling, and inhibiting cell apoptosis. As expected, Nrf2 knockdown nullified the protective effects of 4-OI, resulting in increased ROS generation and apoptosis. Overall, 4-OI inhibited UVB-induced oxidative stress and apoptosis in HaCaT and PIG1 cells, and the protective effect was dependent on Nrf2 activation in some extent.

To further validate the protective effect of 4-OI *in vivo*, mice were exposed to UVB irradiation with or without injection of 4-OI into the tail vein. Our data showed that 4-OI protected the mouse skin from UVB-induced damage. Consistent with our results, a study showed that administration of dimethyl itaconate (DI), another membrane-permeable derivative of itaconate, attenuated skin damage in an imiquimod- (IMQ-) induced psoriasis-like mouse model maintaining intestinal barrier function [35]. Moreover, 4-OI inhibited renal fibrosis and reduced ROS production in an adenine-induced fibrosis rat model. These data illustrate that itaconate, including 4-OI, may be a potential and promising option for treating various ROS-mediated disorders.

## 5. Conclusions

Our study suggests that 4-OI protects melanocytes and keratinocytes from UVB-induced damage by activating the Nrf2/HO-1 pathway, which is one of the possible signaling pathways, to reduce ROS generation and alleviate subsequent apoptosis. In addition, we demonstrated the protective effect of 4-OI on UVB-induced skin damage in an *in vivo* mouse model. Since 4-OI is an endogenous metabolite with low toxicity, it can serve as a potential agent in treating skin disorders related to oxidative stress, such as vitiligo.

## Data Availability

The data generated during and/or analyzed during the current study are available from the corresponding author on reasonable request.

## Supplementary Materials

Supplementary Materials Table 1: primer sequences used for qPCR. Table 2: antibodies used for western blot analysis.
