# Supplementary material for: Four-Octyl Itaconate Attenuates UVB-Induced Melanocytes and Keratinocytes Apoptosis by Nrf2 Activation-Dependent ROS Inhibition
Source: Oxid Med Cell Longev. 2022 Mar 11;2022:9897442. doi: 10.1155/2022/9897442 (PMC8933077; doi:10.1155/2022/9897442)
Supplement: Supplementary Materials — Table 1: primer sequences used for qPCR. Table 2: antibodies used for western blot analysis. [file 9897442.f1.docx]

**Supplementary Table 1. Primer sequences used for qPCR.**

| Primer name | Sequence (5'-3') |
| --- | --- |
| Nrf2 FW | TCAGCGACGGAAAGAGTATGA |
| Nrf2 RV | CCACTGGTTTCTGACTGGATGT |
| HO-1 FW | AAGACTGCGTTCCTGCTCAAC |
| HO-1 RV | AAAGCCCTACAGCAACTGTCG |
| ACTB FW | ATTGCCGACAGGATGCAGA |
| ACTB RV | GAGTACTTGCGCTCAGGAGGA |

**Supplementary Table 2. Antibodies used for Western blot analysis.**

| Antibodies | Product code | Company |
| --- | --- | --- |
| Anti-Keap1 | Ab227828 | Abcam |
| Anti-Nrf2 | ab62352 | Abcam |
| Anti-HO-1 | ab13248 | Abcam |
| Anti-β-Actin | ab8227 | Abcam |
| Goat Anti-Mouse | 115-035-003 | Jackson |
| Goat Anti-Rabbit | 111-035-003 | Jackson |
| iFluor^TM^ 488 antibody | Cat#:16608 | AAT Bioquest |
